# Repetitive Compressive Loading Downregulates the Expression of Autophagy-Related Factors, Autophagy Capacity and Cellular Activity in Human Osteoarthritic Chondrocytes

**DOI:** 10.3390/ijms27167485

**Published:** 2026-08-21

**Authors:** Satomi Sato, Hideaki Iwata, Takeaki Yamamoto, Shu Somemura, Masahiro Takemoto, Yuki Takahashi-Suzuki, Yodo Sugishita, Hiroto Fujiya, Naoki Haraguchi, Kazuo Yudoh

**Affiliations:** 1 Department of Orthopaedic Surgery, St. Marianna University School of Medicine, Sugao 2-16-1, Miyamae ku, Kawasaki 216-8512, Japan; satomi.kimura@marianna-u.ac.jp (S.S.); hideaki.iwata@marianna-u.ac.jp (H.I.); t.yamamoto@marianna-u.ac.jp (T.Y.); masahiro.takemoto@marianna-u.ac.jp (M.T.); naoki.haraguchi@marianna-u.ac.jp (N.H.); 2 Department of Sports Medicine, St. Marianna University School of Medicine, Sugao 2-16-1, Miyamae-ku, Kawasaki 216-8511, Japan; s3somemura@marianna-u.ac.jp (S.S.); fujiya-1487@marianna-u.ac.jp (H.F.); 3 Department of Frontier Medicine, Institute of Medical Science, St. Marianna University School of Medicine, Sugao 2-16-1, Miyamae-ku, Kawasaki 216-8512, Japan; y2takahashi@marianna-u.ac.jp (Y.T.-S.); yodo@marianna-u.ac.jp (Y.S.)

**Keywords:** chondrocytes, osteoarthritis, mechanical stress, autophagy, mitochondria, ATG5, Beclin-1, Parkin

## Abstract

Mechanical stress is thought to be involved in the pathogenesis and pathophysiology of osteoarthritis (OA). However, much remains to be elucidated regarding how chondrocytes sense and respond to mechanical stress (stress sensing and response factors). Additionally, it still remains unclear whether there are defensive responses and mechanisms to protect against pathological agents and mechanical stress in articular cartilage tissue. This study was designed to determine whether repetitive mechanical force, at physiologic levels, affects the expression of factors regulating autophagy such as the autophagy-related proteins ATG5, Beclin-1, and Parkin, and the autophagy process as well as cellular activity in cultured chondrocytes. Three-dimensional cultured tissue was generated from human chondrocytes using a collagen sponge scaffold. After physiological mechanical loading of the 3D cell–collagen sponge construct, comparative analyses of expression levels of ATG5, Beclin-1, and Parkin were performed in human chondrocytes. Chondrocyte activity and Transmission Electron Microscopy (TEM) analysis for detecting autophagy process were also analyzed with or without repetitive compressive loading. In chondrocytes, 60 min or 180 min repetitive compressive loading significantly decreased the expression of ATG5, Beclin-1 and Parkin in comparison with the non-loading group. TEM analysis indicated that, in normal chondrocytes of the non-loading group, the autophagy process was shown to be progressing. In contrast, repetitive loading decreased the number of autophagosomes and autolysosomes in chondrocytes. In addition, numerous degenerated organelles that had not undergone autophagy were observed within the chondrocytes under repetitive loading. The ATG5 and Beclin-1 proteins are known to play crucial roles in regulating cellular autophagy. Furthermore, repetitive mechanical loading caused a decreased expression of Parkin, a mitophagy regulator in chondrocytes. Our results indicate for the first time that a decrease in mitophagy, as well as cellular autophagy, in response to mechanical stress, even at the physiologic level, leads to the accumulation of defective mitochondria and abnormal cellular proteins, resulting in reduced chondrocyte activity and affecting the maintenance of cartilage tissue homeostasis, ultimately contributing to the progression of OA.

## 1. Introduction

Osteoarthritis (OA) is a common, degenerative joint disease mainly affecting the elderly population. It is characterized by a range of joint disorders, including articular cartilage degeneration, synovitis, osteophyte formation and subchondral bone sclerosis/degeneration, leading to joint malfunction, joint pain and restricted mobility, resulting in major impairments in quality of life [1,2,3,4]. In Japan, it is estimated that there are over 25 million patients with radiographically diagnosed OA showing joint deformation on X-ray, and over 12.8 million patients who experience symptoms such as joint pain and functional impairment, with a prevalence rate approximately 10 to 20 times higher than that of rheumatoid arthritis [3,4,5]. There are over 900,000 new OA cases annually in Japan, and the economic loss is estimated to reach 1% of the gross national product, with concerns about a further increase in the number of patients in the future [4,5,6]. OA is a major clinical and socio-economic burden worldwide.

During the development of OA, extrinsic abnormal stresses including mechanical loading, joint injury, obesity and joint instability, or intrinsic stresses, such as aging, genomic/metabolic abnormality and apoptosis, affect normal activities of articular cartilage and subchondral bone tissues and induce homeostatic imbalance of osteochondral joint tissues [1,2,7,8,9]. Of several key risk factors for OA, recent insights have identified mechanical stress as being pivotal in OA pathogenesis, contributing to chondrocyte mitochondrial dysfunction, oxidative stress, apoptosis and extracellular matrix degradation in articular cartilage [8,10,11,12,13,14,15,16]. However, there are many unknowns, such as how chondrocytes sense and respond to mechanical stress that causes OA and contributes to its onset, whether chondrocytes have a defense mechanism against pathological or excessive stress, and how this is related to the mechanism of cartilage degeneration.

We previously found that Sirtuin (Sirt)-1, a mitochondria regulating factor with nicotinamide adenine dinucleotide (NAD)-dependent deacetylase activity, not only activates DNA repair enzymes (APEX2, Ogg1) in OA chondrocytes but also regulates the activity of the runt-related transcription factor 2 (Runx2), which is involved in hypertrophic chondrogenesis, suggesting that Sirt-1 regulates DNA damage repair, osteophyte formation, energy metabolism and the production of cartilage matrix-degrading enzymes, thus contributing to the pathogenesis of OA [7,9,17,18]. In addition, we demonstrated that excessive reactive oxygen species (ROS), which are leaked from the mitochondrial electron transport chain in response to mechanical stress, contribute to DNA damage and mitochondrial dysfunction in chondrocytes [9,19]. Our findings indicate that mitochondrial function in chondrocytes changes in response to OA-inducing factors including mechanical stress [7,9,18]. Indeed, our reports and those of others provide evidence supporting the idea that chondrocyte energy metabolism—glucose uptake and adenosine triphosphate (ATP) production—temporarily increases in response to mechanical stress such as weight bearing, leading to increased activity of the citric acid cycle and electron transport chain in mitochondria [9,10,11,12,13,14,15,19,20,21]. This results in excessive production and leakage of ROS in mitochondria, ultimately promoting the progression of OA through oxidative stress.

More recently, we analyzed whether continuous or repetitive physiological loading stress may affect chondrocyte activity through changes in mitochondrial function in response to mechanical loading [22]. The results confirmed that repetitive mechanical loading on articular cartilage promotes the expression of the mitochondrial regulator Sirt-1, and that mitochondrial NAD is consumed during the Sirt-1 activation process, resulting in a simultaneous decrease in NAD activity, and, consequently, a decrease in mitochondrial activity and ATP production in chondrocytes. Furthermore, our study revealed that activation of Sirt-1 by repetitive loading induces expression of the osteogenic and hypertrophic chondrogenic transcription factor Runx2 in chondrocytes, since Sirt-1 is known to positively regulate the activity of Runx2. In addition, since Runx2 is recognized as a promoter of matrix metalloproteinase (MMP)-13, repetitive loading may enhance production of the OA-related factor MMP-13 through the mechanical stress-induced Sirt-1–Runx2 pathway [22]. Also, numerous reports have demonstrated that mitochondrial dysfunction is correlated with cell damage and a wide range of age-related diseases, including OA [23,24,25,26,27,28].

**Figure 1:**
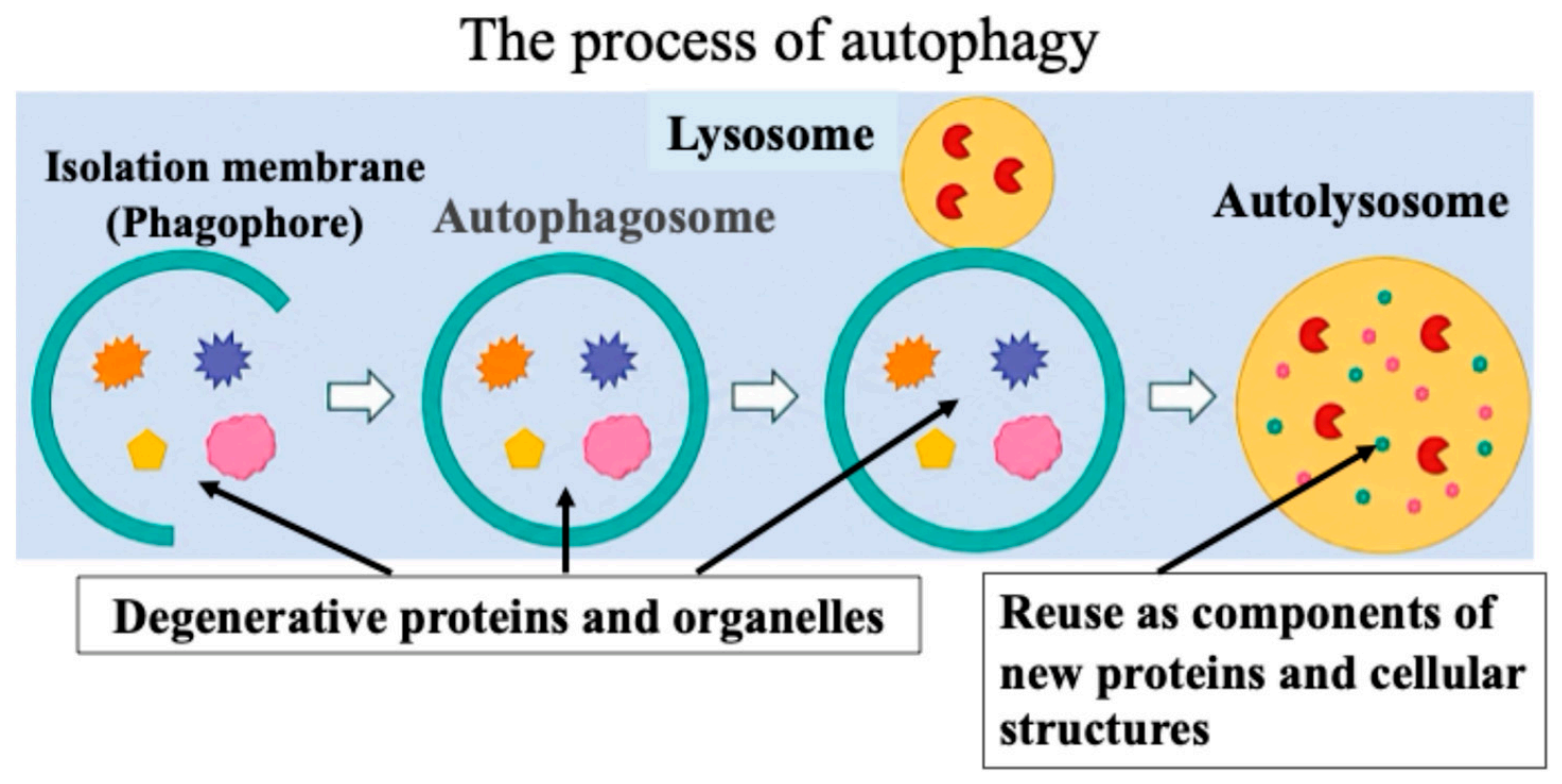
Process of autophagy. Autophagy is a series of processes that enzymatically break down and reuse unwanted substances within cells. Autophagosomes are spherical, double-membrane structures that enclose waste products. Defective mitochondria, abnormal proteins, and damaged organelles taken up by autophagosomes are degraded by lysosomes. The substances degraded by enzymes become small molecules such as amino acids, which are reused as new proteins and cell components.

Recently, the implications of chondrocyte autophagy (self-eating) in the pathogenesis of OA have attracted attention [29,30,31,32,33,34,35,36,37]. Autophagy maintains intracellular energy homeostasis and protects cells against various stress conditions, such as inflammation, oxidative stress and mechanical stress [30,31]. Degenerative proteins and damaged mitochondria are recognized by the autophagy mechanism through the emission of specific signals. As shown in Figure 1, the next process is the formation of an autophagosome, a spherical double-membrane structure, that encapsulates waste products. The autophagosome then fuses with a lysosome. Lysosomes are organelles containing powerful digestive enzymes called hydrolytic enzymes. Lysosomes break down the waste products trapped within the autophagosome. The enzymatic degraded substances become small molecules such as amino acids, which can be used as building blocks for new proteins and cellular components. Autophagy is a rational system by which cells break down and reuse their own components (Figure 1) [31,32,33,34,35].

In addition to the autophagy of intracellular components, mitophagy, a specialized form of autophagy that functions in regulating the turnover of dysfunctional mitochondria, acts as a pivotal mechanism for mitochondrial homeostasis [29,34,35,36,37,38]. Mitophagy impairment gives rise to the progressive accumulation of defective mitochondria, leading to chondrocyte apoptosis and cartilage matrix degradation, consequently contributing to the degeneration of cartilage [38].

Several reports have confirmed that chondrocytes in OA have reduced autophagy capacity compared to normal chondrocytes, suggesting that an increase in chondrocytes with accumulated dysfunctional mitochondria in OA cartilage may be involved in the onset of OA [29,30,31,32,33,34,35,36,37]. Chondrocytes in articular cartilage may possess defense mechanisms such as autophagy against cellular damage and mitochondrial defects associated with OA-related factors, namely, mechanisms that selectively degrade defective mitochondria, that are no longer able to produce ATP, through mitophagy as well as degrading abnormal proteins through intracellular autophagy. However, in addition to the importance of understanding the changes in chondrocyte mitochondrial function induced by the mechanical stress response, many aspects remain unclear regarding the relationship between changes in the autophagic capacity to destroy damaged cellular proteins/mitochondria due to mechanical stress, the resultant accumulation of damaged cells or mitochondria, and the mechanism of cartilage degeneration.

Numerous reports have demonstrated the molecular pathway of autophagy in a variety of cells [30,31,39,40,41,42]. The autophagy-related protein, ATG5, is required for cellular autophagy. Cellular autophagy is a catabolic process that transports large amounts of cytoplasmic contents to the autophagosome–lysosome for degradation (Figure 1). ATG5 plays an important role in autophagy as well as in the apoptotic process, likely through modifying the cytoskeleton [38,43,44]. Beclin-1 is also known to be a protein involved in autophagy induction. Beclin-1 interacts with various autophagy-related cofactors to form a complex inducing autophagy [45,46]. In contrast to ATG5 and Beclin-1, Parkin has been shown to induce the autophagic degradation (mitophagy) of damaged mitochondria: (1) Parkin degrades various outer membrane proteins of depolarized mitochondria; (2) as a result, the outer mitochondrial membrane can be disrupted; and (3) proteasomes localize to the depolarized outer mitochondrial membrane, causing proteolysis and disruption of the outer mitochondrial membrane [47,48,49,50]. Based on these studies, we hypothesized that a decrease in mitophagy, as well as cellular autophagy, in response to mechanical stress leads to the accumulation of defective mitochondria and abnormal cellular proteins, resulting in reduced chondrocyte activity and affecting the maintenance of cartilage tissue homeostasis, ultimately contributing to the progression of OA.

In the current study, we focused on the effect of physiologic mechanical loading on the expression of three autophagy-relating factors, ATG5, Beclin-1 and Parkin, in OA chondrocytes. Using an experimental OA model with repeated weight-bearing, we investigated the involvement of these three autophagy-related factors in the mechanism of cartilage degeneration. For the first time, we have shown that even at a physiological level, repetitive mechanical stress reduces autophagy activity that targets both dysfunctional cellular proteins and dysfunctional mitochondria, thereby suppressing chondrocyte activity in articular cartilage.

## 2. Results

### 2.1. Conditions for Repetitive Compressive Loading of the 3D Cell-Culture Construct

**Figure 2:**
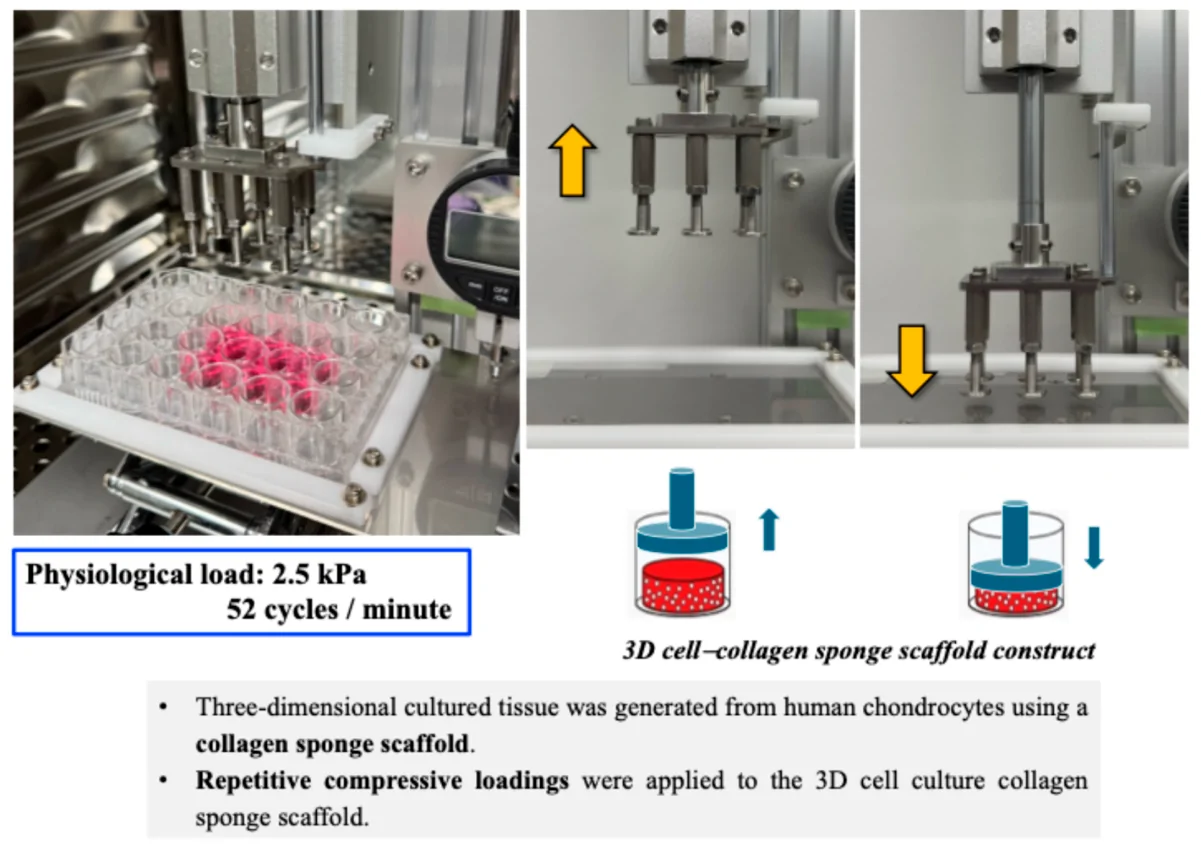
Repetitive compressive loading was applied to the 3D cell-culture collagen sponge scaffold at 0 kPa (non-loaded) or 25.5 gf/cm^2^ (2.5 kPa) × 52 cycles/min for 60 min or 180 min. (Representative images of generation of the 3D cell–collagen scaffold construct for human cultured cells. Scheme showing mechanical loading of the 3D cell-culture construct.).

Figure 2 shows the set-up used to apply repetitive physiologic compressive loading to the 3D cell–collagen sponge constructs. Our preliminary experiments and previous reports confirmed that a physiological load does not induce cell damage or cell death (apoptosis), and so all subsequent experiments were carried out at 25.5 gf/cm^2^ (2.5 kPa) as a physiologic level of mechanical loading [22].

Based on evaluations conducted during the preparatory design phase, the characteristics of the testing apparatus are described as follows:

- The apparatus applied a sinusoidal compressive load with a peak of 2.5 kPa at a frequency of approximately 0.87 Hz, resulting in a compressive strain in the range of a few per mille to just under 1%.
- The cycle repeated every 1.15 s, corresponding to the rhythm of single-leg weight-bearing stress observed during a heartbeat or slow walking; consequently, a rate of 52 cycles per min (0.87 Hz) was selected as the test condition.
- Repetitive load performances (torque, cycle repeatability, speed) and their adjustability were analyzed after 15 min, 30 min, 1 h, 6 h, 12 h, 24 h, and 72 h of operation, and it was confirmed that their errors at each time point were less than 0.5%.

Regarding stress levels, we sought to establish a load—mimicking the repetitive stress of a walking rhythm that would not immediately induce cell death or reduce cell viability in the 3D collagen sponge cartilage constructs used in this study; we confirmed that a range of 2.5–4.0 kPa did not adversely affect cell viability. This level is consistent with findings from the previously published literature [22] and is further supported by data obtained from in vitro cell viability assessments (CCK-8 assay). No significant differences in the cell viability (CCK-8 assay) were observed between the loading group and the control group, as shown in Supplementary Data.

### 2.2. Effect of Physiologic and Repetitive Compressive Loading on Cell Protein Content in a 3D Cell-Culture Construct After Mechanical Loading

**Figure 3:**
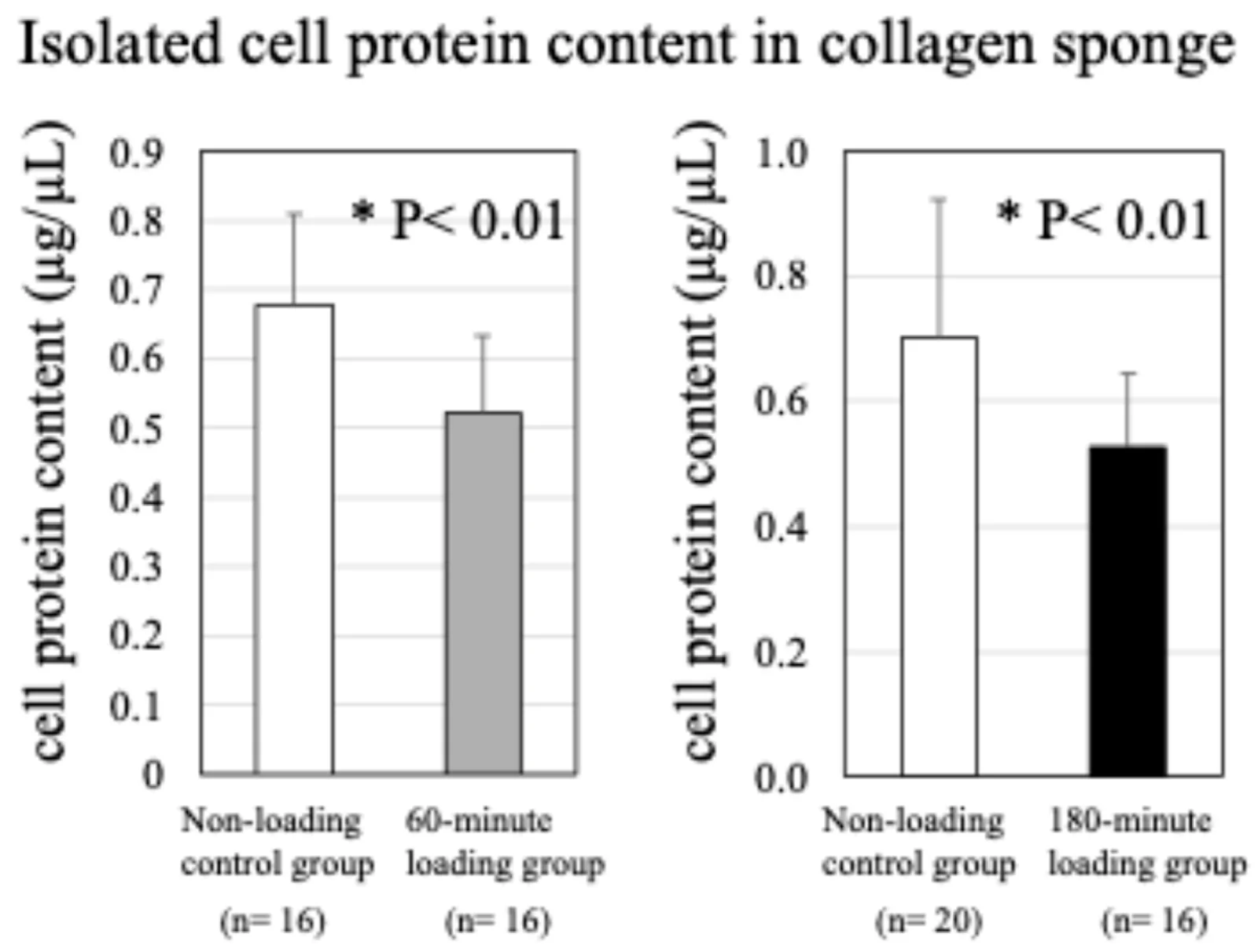
Effects of physiologic and repetitive compressive loading on cell protein content in the 3D chondrocyte-culture construct after mechanical loading. The application of repetitive and physiologic mechanical stress for 60 or 180 min resulted in a significant decrease in cell protein content between the non-loading control group and the repetitive loading groups (control vs. 60 min loading: * *p* < 0.01, control vs. 180 min loading: * *p* < 0.01).

As shown in Figure 3, repetitive compressive loading resulted in a significant decrease in the extracted cell protein concentration from the 3D cell-culture construct after both 60 min and 180 min mechanical loading, as compared to the respective non-loading control (control (*n* = 16) vs. 60 min loading (*n* = 16): *p* < 0.01, control (*n* = 20) vs. 180 min loading (*n* = 16): *p* < 0.01, Figure 3).

### 2.3. Transmission Electron Microscopy (TEM) Analysis

**Figure 4:**
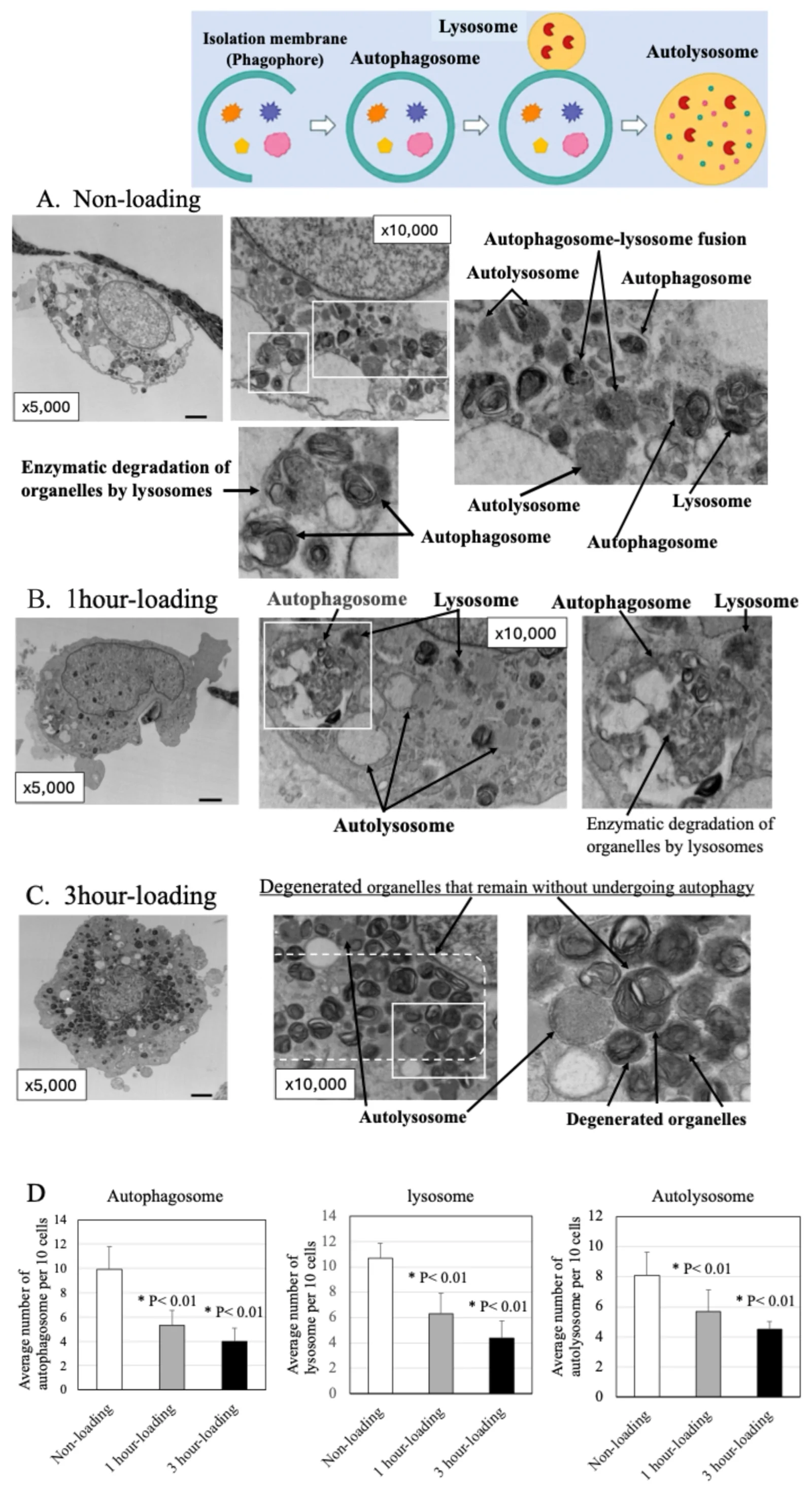
Transmission Electron Microscopy (TEM) analysis. (**A**): In chondrocytes of the non-loading group, a large number of autophagosomes and lysosomes showing the autophagy process, as well as autolysosomes after digestion of organelles, were observed. (**B**): In chondrocytes of the 1 h loading group, although fewer in number than in the no-load group, autophagosomes, lysosomes, and autolysosomes were seen indicating that the autophagy process was observed. (**C**): In chondrocytes of the 3 h loading group, the number of autophagosomes and autolysosomes was lower compared to the no-load and 1 h loading groups. On the other hand, there were numerous degenerated organelles that had not undergone autophagy within the chondrocytes. This suggests the accumulation of defective mitochondria and abnormal cellular proteins within the cells. (**D**): The numbers of autophagosomes, lysosomes, and autolysosomes per cell were significantly lower in mechanical loading groups than in non-loading group (* *p* < 0.01).

As shown in representative TEM images in Figure 4A, in the non-loading group, numerous autophagosomes, lysosomes, and autophagosome–lysosome fusion exhibiting the autophagy process, as well as autolysosomes following the digestion of organelles, were observed in chondrocytes.

In the chondrocytes of the 1 h loading group (Figure 4B), autophagosomes, lysosomes, and autolysosomes, which indicate the autophagy process, were observed, although in smaller numbers than in the chondrocytes of the non-loading group.

In the 3 h loading group, the number of autophagosomes and autolysosomes in chondrocytes was lower compared to the no-loading and 1 h loading groups, while a large number of degenerated organelles that had not undergone autophagy were observed in chondrocytes (Figure 4C).

The numbers of autophagosomes, lysosomes, and autolysosomes per cell were significantly lower in the 1 h loading group and the 3 h loading group in comparison with the control (*p* < 0.01, Figure 4D). Regarding the size of these organelles, there were no significant differences among subgroups.

### 2.4. Effects of Physiologic and Repetitive Compressive Loading on the Levels of the Autophagy-Related Protein ATG5 in Chondrocytes

**Figure 5:**
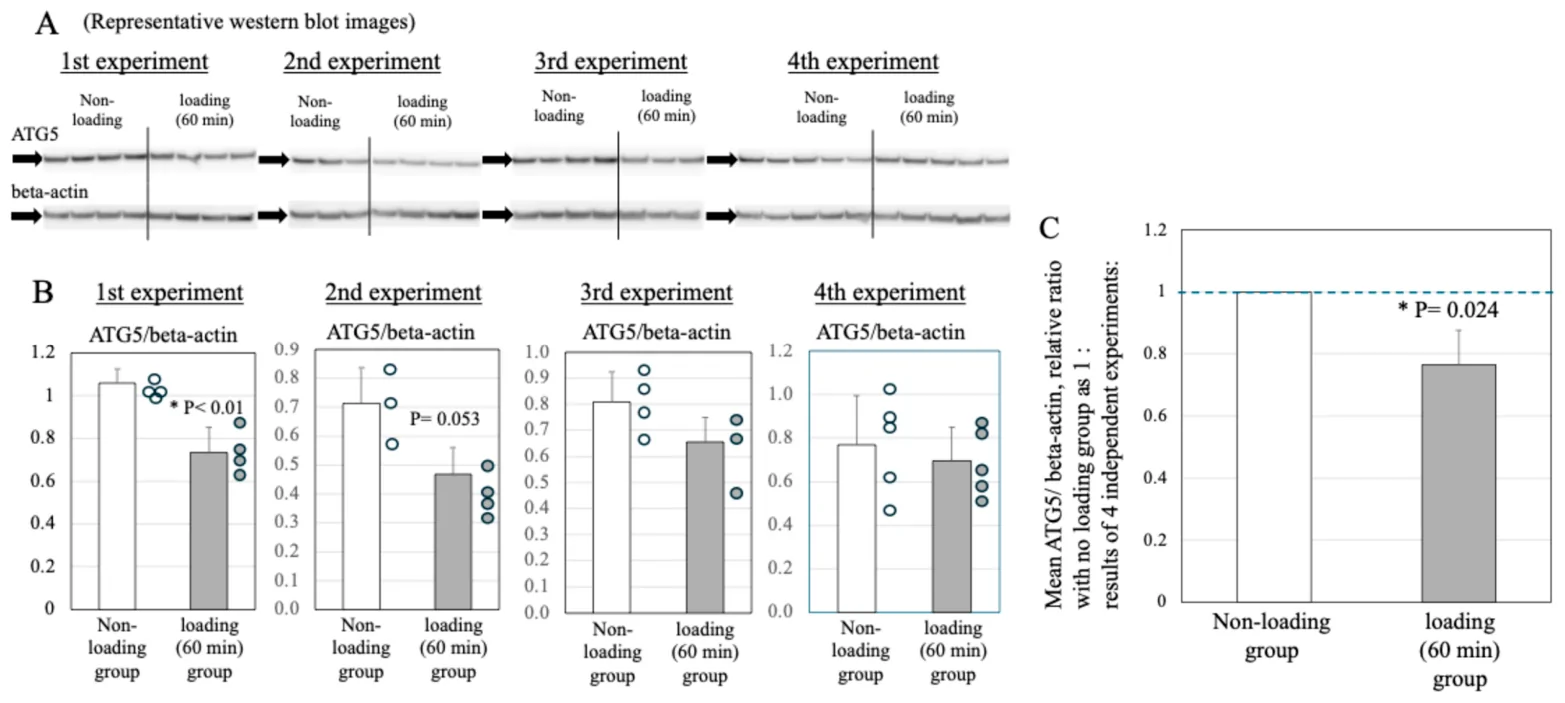

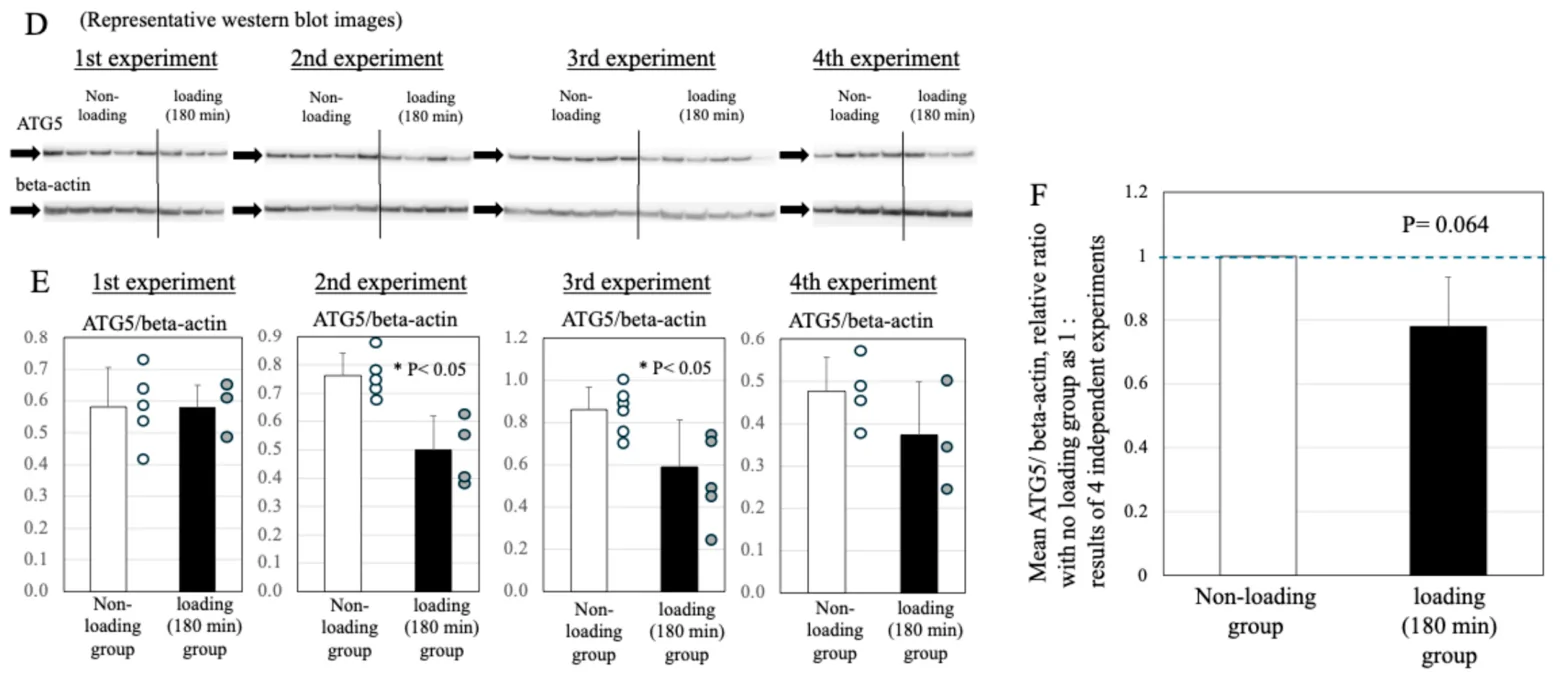
Effects of physiologic and repetitive compressive loading on the expression of ATG5 in chondrocytes. (**A**): Expression of ATG5 in non-loaded chondrocytes and 60 min loaded chondrocytes (representative Western blotting images: 1st, 2nd, 3rd, 4th experiment). (**B**): Mean ratio relative to beta-actin. ATG5 protein levels showed a tendency to decrease in 60 min loaded chondrocytes compared to non-loaded chondrocytes in the 1st experiment (* *p* < 0.01) and the 2nd experiment (*p* = 0.053). (**C**): Results of four independent experiments: mean ATG5 levels/beta-actin levels, relative ratio with the non-loading group set as 1. Treatment for 60 min with repetitive compressive loading significantly downregulated the expression of ATG5 in chondrocytes in comparison with the control (*n* = 4 independent experiments, control vs. 60 min loading: * *p* = 0.024). (**D**): Expression of ATG5 in non-loaded chondrocytes and 180 min loaded chondrocytes (representative Western blotting images: 1st, 2nd, 3rd, 4th experiment). (**E**): Mean ratio relative to beta-actin. ATG5 protein levels showed a decreasing trend in 180 min loaded chondrocytes compared to non-loaded chondrocytes in the 2nd and 3rd experiments (* *p* < 0.05). (**F**): Results of four independent experiments: mean ATG5 levels/beta-actin levels relative ratio with the non-loading group set as 1. Treatment for 180 min with repetitive compressive loading did not influence the expression of ATG5 in chondrocytes in comparison with the control (*n* = 4 independent experiments, control vs. 180 min loading: *p* = 0.064). White circles indicate the data scatter plot to the figure legend (**B**,**E**).

Decreased ATG5 expression in chondrocytes was directly related to repetitive compressive loading (*n* = 4/experiment, *n* = 4 independent experiments, control vs. 60 min-loading: 1st experiment (*p* < 0.05), 2nd experiment (*p* = 0.053), Figure 5A,B). Treatment for 60 min with repetitive compressive loading significantly down-regulated the expression of ATG5 in chondrocytes compared to the control (*n* = 4 independent experiments, control vs. 60 min-loading: *p* = 0.024, Figure 5C).

Treatment for 180 min with repetitive compressive loading down-regulated the expression of ATG5 in chondrocytes compared to the non-loaded control (*n* = 4/experiment, control vs. 180 min-loading: 2nd and 3rd experiment (*p* < 0.05), Figure 5D,E; *n* = 4 independent experiments, control vs. 180 min-loading (*p* = 0.064), Figure 5F).

### 2.5. Effect of Physiologic and Repetitive Compressive Loading on the Levels of the Autophagy-Related Protein Beclin-1 in Chondrocytes

**Figure 6:**
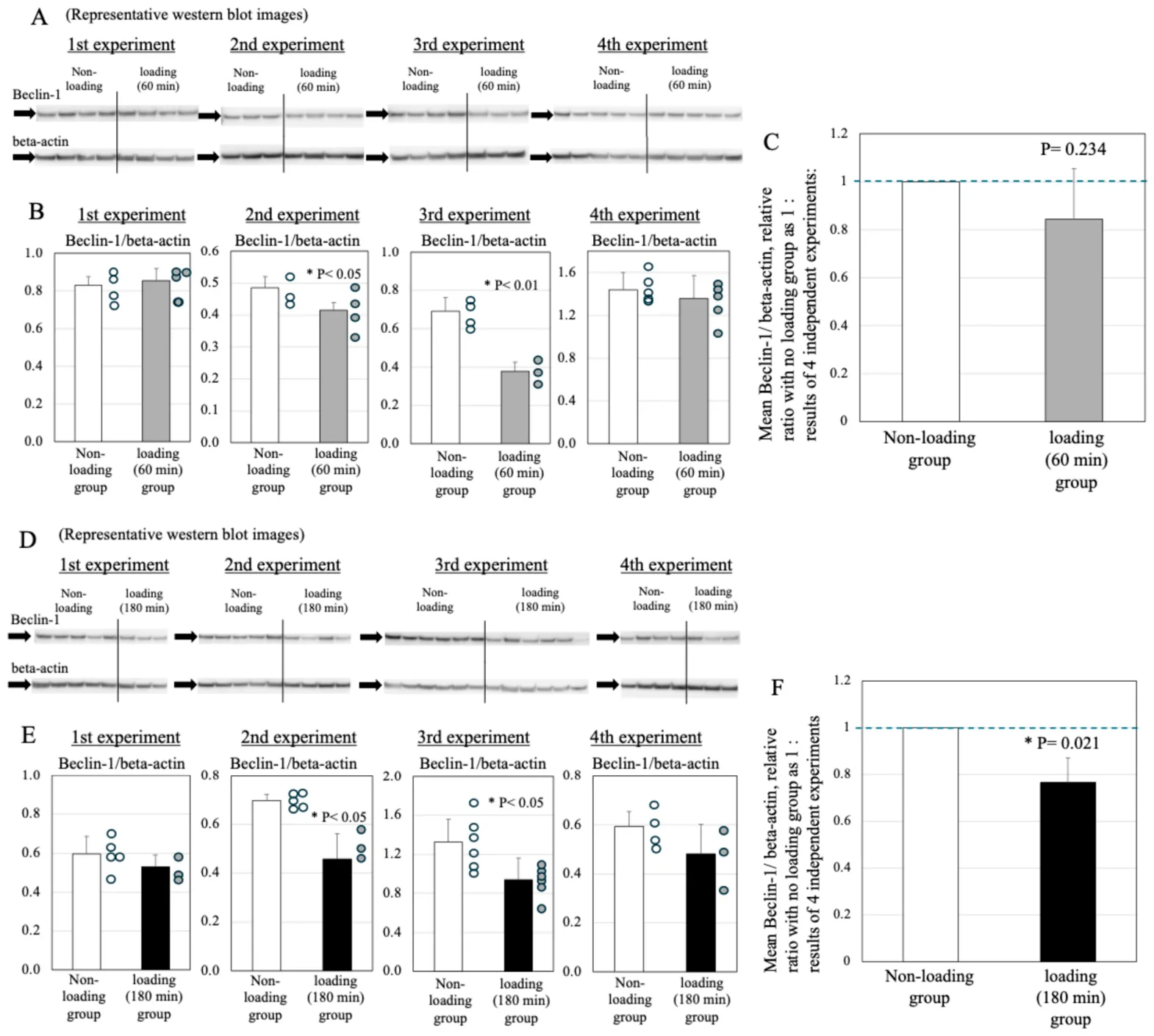
Effects of physiologic and repetitive compressive loading on the expression of Beclin-1 in chondrocytes. (**A**): Expression of Beclin-1 in non-loaded chondrocytes and 60 min loaded chondrocytes (representative Western blotting images: 1st, 2nd, 3rd, 4th experiment). (**B**): Mean ratio relative to beta-actin. Beclin-1 protein levels showed a decreasing trend in 60 min loaded chondrocytes compared to non-loaded chondrocytes [2nd experiment (* *p* < 0.05), 3rd experiment (* *p* < 0.01)]. (**C**): Results of four independent experiments: mean Beclin-1 levels/beta-actin levels relative ratio with the non-loading group set as 1. Treatment for 60 min with repetitive compressive loading showed no significant change in the expression of ATG5 in chondrocytes in comparison with the control (*n* = 4 independent experiments, control vs. 60 min loading: *p* = 0.234). (**D**): Expression of Beclin-1 in non-loaded chondrocytes, 180 min loaded chondrocytes (representative Western blotting images: 1st, 2nd, 3rd, 4th experiment). (**E**): Mean ratio relative to beta-actin. Beclin-1 protein levels showed a decreasing significantly decreased in 180 min loaded chondrocytes compared to non-loaded chondrocytes in the 2nd and the 3rd experiments (* *p* < 0.05). (**F**): Results of four independent experiments: mean Beclin-1 levels/beta-actin levels relative ratio with the non-loading group set as 1. Treatment for 180 min with repetitive compressive loading significantly decreased the expression of Beclin-1 in chondrocytes in comparison with the control (*n* = 4 independent experiments, control vs. 180 min loading: * *p* = 0.021). White circles indicate the data scatter plot to the figure legend (**B**,**E**).

As shown in Figure 6, treatment with repetitive compressive loading caused a decrease in the levels of Beclin-1 expression in chondrocytes, compared with the control in each experiment [*n* = 4/experiment, *n* = 4 independent experiments, control vs. 60 min-loading: 2nd and 3rd experiment (*p* < 0.05), Figure 6A,B]. No significant difference was observed in the Beclin-1 expression between the non-loading control and 60 min loading group (*n* = 4 independent experiments, control vs. 60 min-loading: *p* = 0.234, Figure 6C).

Meanwhile, treatment for 180 min with repetitive compressive loading significantly down-regulated the expression of Beclin-1 in chondrocytes compared to the control [*n* = 4 independent experiments, control vs. 180 min-loading: 2nd and 3rd experiment (*p* < 0.05), Figure 6D,E; *n* = 4 independent experiments, control vs. 180 min-loading (*p* = 0.021), Figure 6F].

### 2.6. Effects of Physiologic and Repetitive Compressive Loading on Levels of the Autophagy-Related Protein Parkin in Chondrocytes

**Figure 7:**
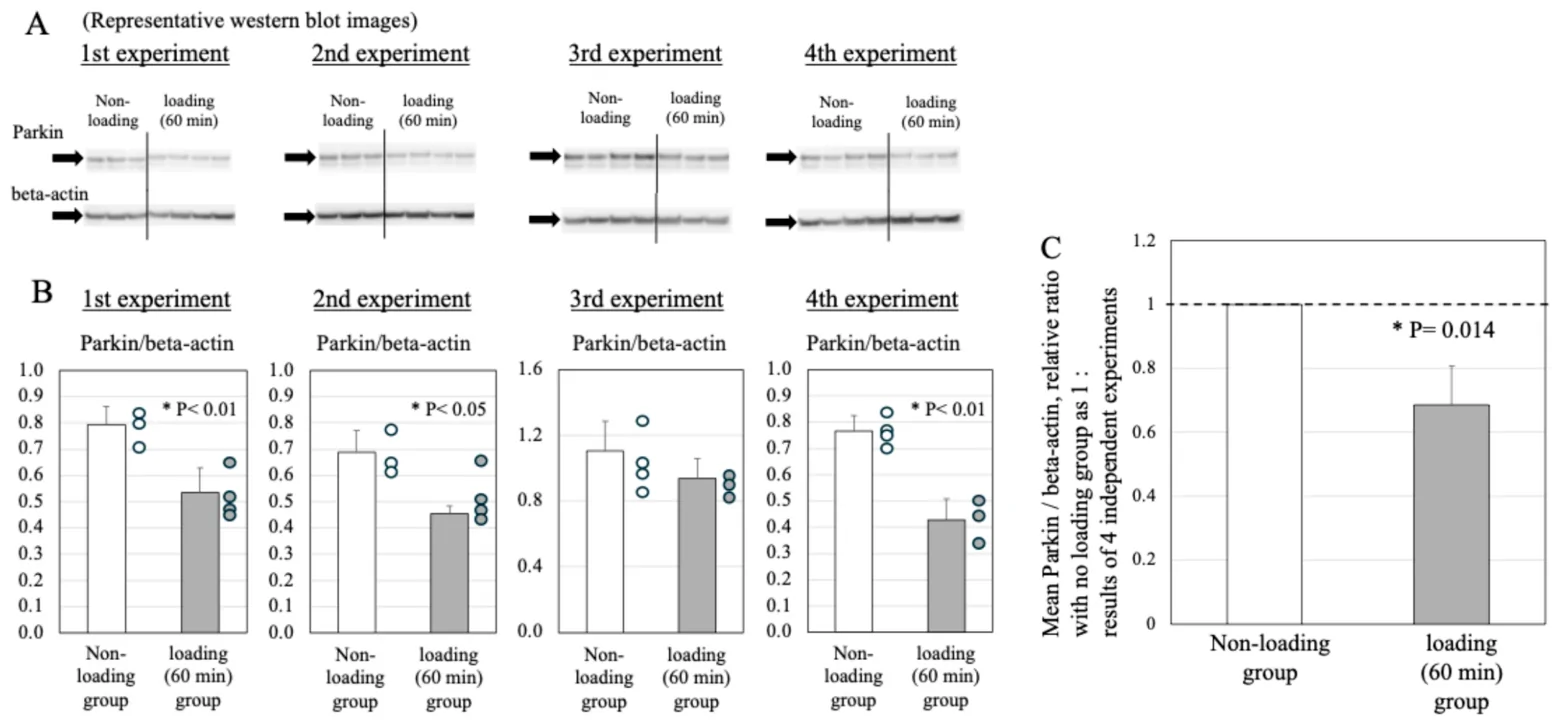

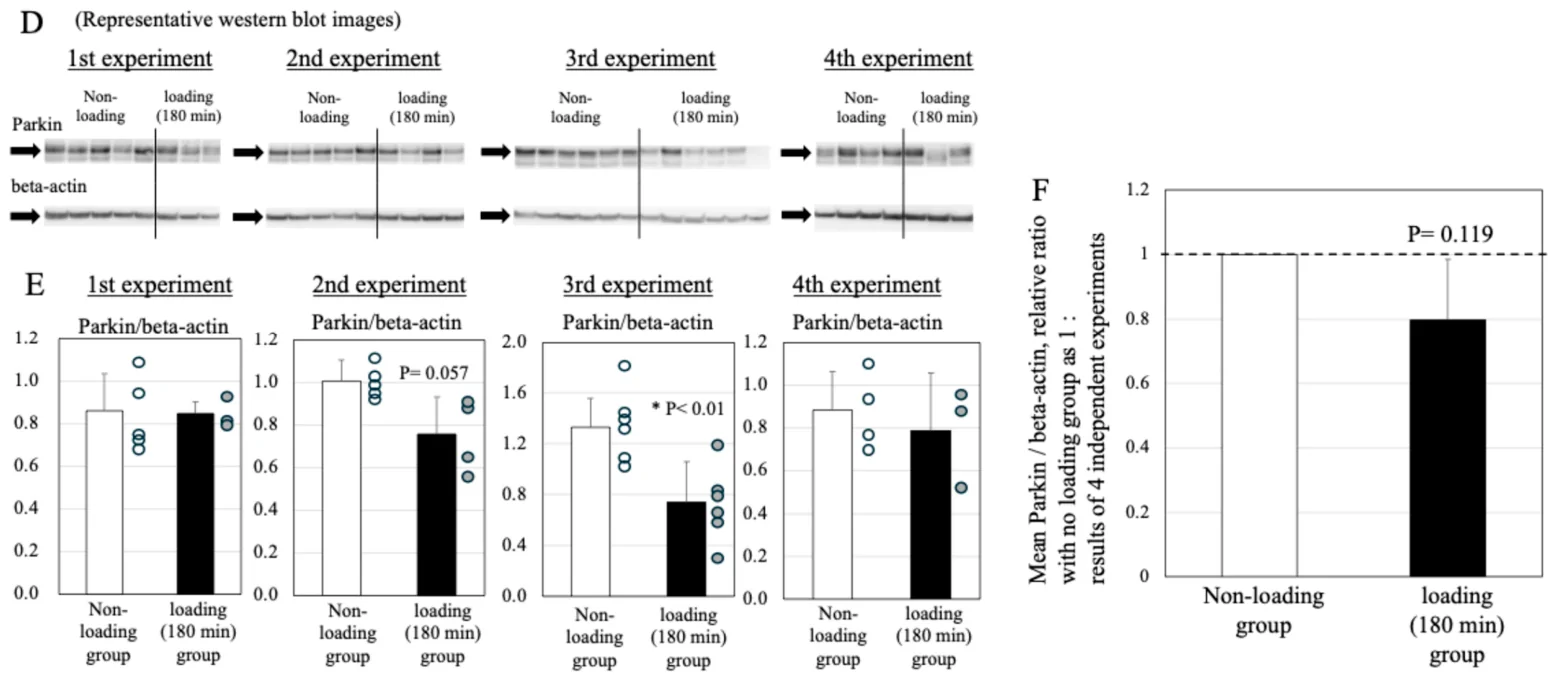
Effects of physiologic and repetitive compressive loading on the expression of Parkin in chondrocytes. (**A**): Expression of Parkin in non-loaded chondrocytes and 60 min loaded chondrocytes (representative Western blotting images: 1st, 2nd, 3rd, 4th experiment). (**B**): Mean ratio relative to beta-actin. Parkin protein levels showed a decreasing trend in 60 min loaded chondrocytes compared to non-loaded chondrocytes [1st experiment (* *p* < 0.01), 2nd experiment (* *p* < 0.05), 4th experiment (* *p* < 0.01)]. (**C**): Results of four independent experiments: mean Parkin levels/beta-actin levels relative ratio with the non-loading group set as 1. Treatment for 60 min with repetitive compressive loading significantly decreased the expression of Parkin in chondrocytes in comparison with the control (*n* = 4 independent experiments, control vs. 60 min loading: * *p* = 0.014). (**D**): Expression of Parkin in non-loaded chondrocytes and 180 min loaded chondrocytes (representative Western blotting images: 1st, 2nd, 3rd, 4th experiment). (**E**): Mean ratio relative to beta-actin. Parkin protein levels showed a decreasing trend in 180 min loaded chondrocytes compared to non-loaded chondrocytes [2nd experiment (*p* = 0.057), 3rd experiment (* *p* < 0.01)]. (**F**): Results of four independent experiments: mean Parkin levels/beta-actin levels relative ratio with the non-loading group set as 1. Treatment for 180 min with repetitive compressive loading did not influence the expression of Beclin-1 in chondrocytes in comparison with the control (*n* = 4 independent experiments, control vs. 180 min loading: *p* = 0.119). White circles indicate the data scatter plot to the figure legend (**B**,**E**).

The repetitive compressive loading groups showed downregulation of the expression of Parkin in chondrocytes in comparison to the non-loading control group in each experiment [*n* = 3–4/experiment, *n* = 4 independent experiments, control vs. 60 min-loading: 1st, 2nd, and 4th experiment (*p* < 0.05), Figure 7A,B]. There was a significant difference in the Parkin level in chondrocytes between the non-loading control and 60 min loading group (*n* = 4 independent experiments, control vs. 60 min loading: *p* = 0.014, Figure 7C).

In the treatment for 180 min with repetitive compressive loading, no significant difference in the expression of Parkin was observed between the non-loading control and 180 min loading group [*n* = 4/experiment, control vs. 180 min loading: 2nd experiment (*p* = 0.057), 3rd experiment (*p* < 0.05), Figure 7D,E; *n* = 4 independent experiments, control vs. 180 min loading (*p* = 0.119), Figure 7F].

### 2.7. Effect of Physiologic and Repetitive Compressive Loading on Chondrocyte Activities, Type II Collagen and Proteoglycan Production, by Chondrocytes

**Figure 8:**
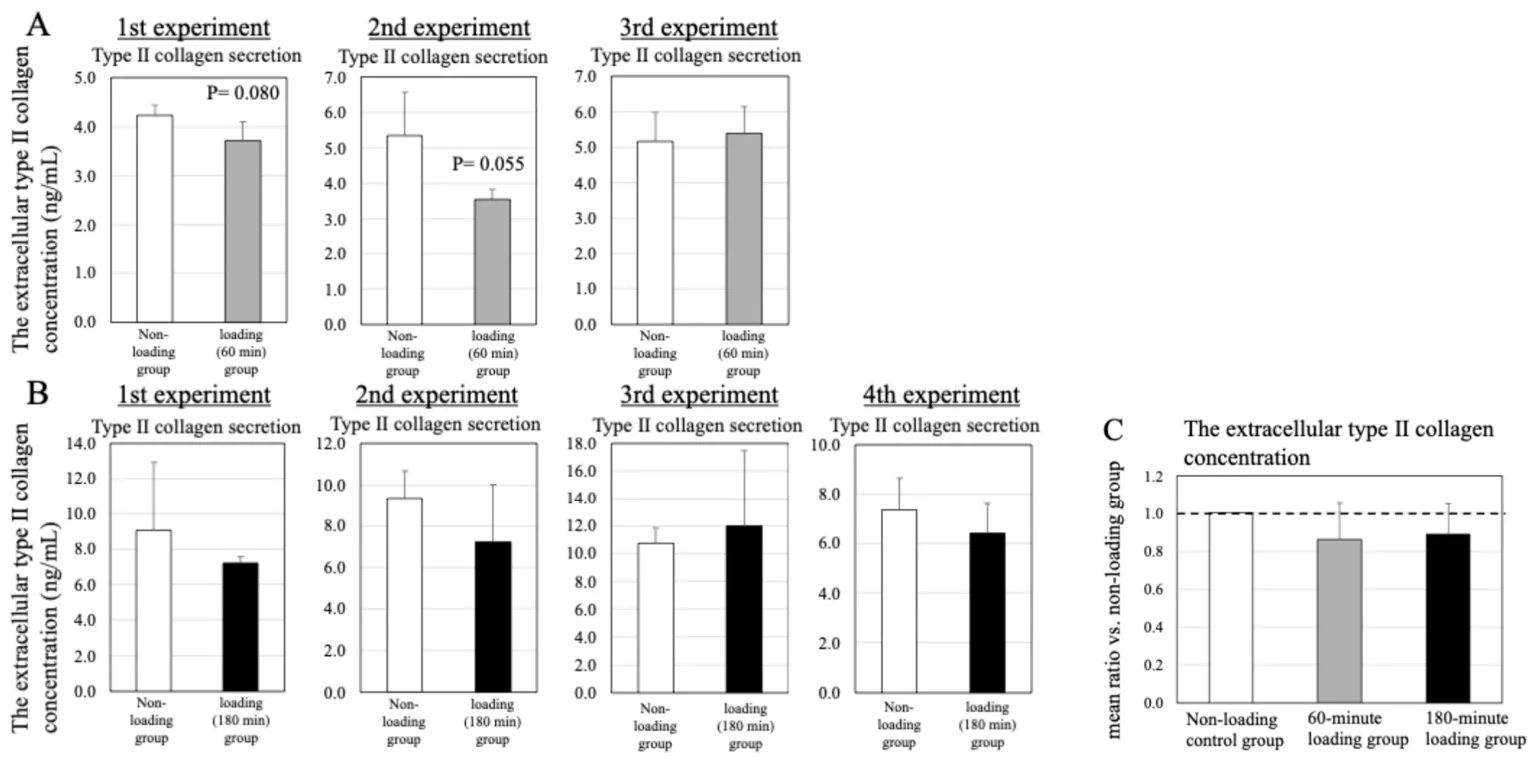
Effect of physiologic and repetitive compressive loading on type II collagen production by chondrocytes. (**A**): The application of repetitive and physiologic mechanical stress for 60 min decreases the production of type II collagen by chondrocytes in comparison with the control [1st experiment (*p* = 0.080), 2nd experiment (*p* = 0.055)]. (**B**): Repeated compressive loading of chondrocytes for 180 min did not result in significant differences in type II collagen production compared with non-loaded controls. (**C**): Results of four independent experiments: mean type II collagen production relative expression compared with the non-loading group set as 1.

As shown in Figure 8A, repetitive compressive loading for 60 min tended to suppress the production of type II collagen by 3D-cultured chondrocytes as compared to the non-loading control [*n* = 3/experiment, 3 independent experiments; control vs. 60 min loading: 1st experiment (*p* = 0.080), 2nd experiment (*p* = 0.055), Figure 8A,C]. Repeated compressive loading for 180 min did not result in significant differences in type II collagen production by 3D-cultured chondrocytes compared with unloaded controls [*n* = 4/experiment, 4 independent experiments, Figure 8B,C].

**Figure 9:**
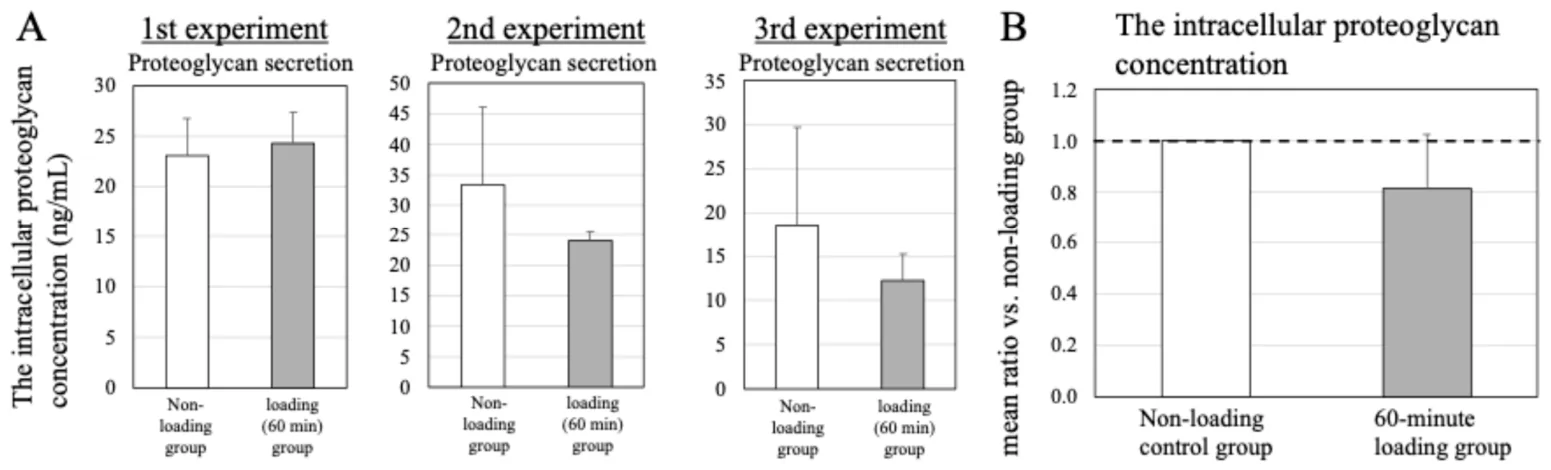
Effect of physiologic and repetitive compressive loading on the level of intracellular proteoglycan in chondrocytes. (**A**): The level of intracellular proteoglycan in chondrocytes showed a decreasing trend in 60 min loaded chondrocytes compared to non-loaded chondrocytes. (**B**): Results of three independent experiments: mean relative ratio with the non-loading group set as 1. There was no significant difference in the proteoglycan production between the non-loading control and the loading group.

Repetitive compressive loading for 60 min tended to decrease the level of intracellular proteoglycan in 3D-cultured chondrocytes compared to the non-loading control [*n* = 3 independent experiments, 3 independent experiments, Figure 9A,B].

The concentration of intracellular proteoglycan in 3D-cultured chondrocytes could not be measured in the repetitive compressive loading for 180 min group, as intracellular proteoglycan concentrations in chondrocytes were below the detection limit.

## 3. Discussion

Autophagy and mitophagy are processes that selectively remove damaged or dysfunctional cellular proteins and mitochondria via the autophagic machinery and that reuse cellular own components, thus acting to maintain the quality control and homeostasis of cells and mitochondria. Our present study indicates that repetitive mechanical loading reduces the expression of the autophagy-regulating factors ATG5, Beclin-1 and Parkin in OA chondrocytes in a 3D chondrocyte-culture system. Since the ATG5 and Beclin-1 proteins are known to play crucial roles in regulating cellular autophagy, repetitive mechanical stress may affect the autophagy of damaged cell organelles, consequently inducing the accumulation of abnormal cellular protein into chondrocytes. Our findings of repetitive mechanical stress-induced downregulation of both ATG5 and Beclin-1 in chondrocytes clearly demonstrate that repetitive mechanical loading, even at a physiologic level, may induce a decrease in cellular autophagic capacity and the resultant accumulation of abnormal cellular proteins, leading to an imbalance in chondrocyte homeostasis. Furthermore, repetitive mechanical loading caused the reduced expression of Parkin, a mitophagy regulator, in chondrocytes, suggesting a potential reduction in chondrocyte mitophagy. Parkin has been shown to induce the autophagic degradation (mitophagy) of damaged mitochondria [50]. Mitochondrial dysfunction is involved in aging and multiple degenerative diseases, including OA. Mitophagy, a process that selectively clears damaged or dysfunctional mitochondria through autophagic machinery, functions to maintain mitochondrial quality control and homeostasis [51].

Indeed, our present TEM analysis indicated that, in chondrocytes of the non-loading group, numerous autophagosomes, lysosomes, autophagosome–lysosome fusion exhibiting the autophagy process, as well as autolysosomes, were observed, suggesting that the autophagic function, in which cellular waste products are enzymatically degraded into small molecules such as amino acids and utilized as components of new proteins and cellular structures, is functioning normally in normal chondrocytes of the non-loading group. In contrast, repetitive loading decreased the number of autophagosomes and autolysosomes in chondrocytes. In addition, numerous degenerated organelles that had not undergone autophagy were observed within the chondrocytes under repetitive loading, suggesting the accumulation of defective mitochondria and abnormal cellular proteins within the cells. It is well known that defective mitochondria, abnormal proteins, and damaged organelles taken up by autophagosomes are broken down by lysosomes and that the substances broken down by enzymes become small molecules such as amino acids, which are reused as new proteins and cellular components (Figure 1). Our results from the TEM analysis indicate that repetitive mechanical stress, even at the physiologic level, may reduce the autophagy process that break down and reuse unwanted substances within cells.

Our present study indicates that a decrease in mitophagy, as well as cellular autophagy, in response to repetitive mechanical stress, leads to the accumulation of both defective mitochondria and abnormal cellular proteins, resulting in reduced chondrocyte activity and affecting cartilage tissue homeostasis, ultimately contributing to the progression of OA. We have previously elucidated the mechanism by which repeated mechanical stress reduces mitochondrial metabolism and cellular activity in OA chondrocytes [22]. With respect to mitochondrial metabolism, we have focused on two key regulators of mitochondrial function, NAD+ and sirtuin 1. Generally, 30–70% of intra-cellular NAD+ is found in the mitochondria [52,53,54,55]. It is well known that NAD+ has a role as a coenzyme in cellular ATP production by mitochondria and that it plays a role as a master regulator of mitochondrial energy metabolism [54]. As with NAD+, sirtuin 1, a longevity gene-related protein, also plays an important role as a mitochondrial regulator acting as an energy sensor during ATP production by mitochondria [50,54,56]. Sirtuin protein is known to be an NAD-dependent deacetylase, and NAD+ is required as a coenzyme for its activation [55,57]. Thus, along with sirtuin activation, NAD+ is consumed and the level of NAD+ in the mitochondria is decreased. Indeed, our previous study demonstrated that repetitive mechanical loading-induced sirtuin 1 activation requires the consumption of NAD+ from mitochondria and leads to a declining trend of NAD+ in the mitochondria of chondrocytes. Subsequently, since NAD+ acts as a coenzyme in ATP production by mitochondria, the NAD+ reduction by repetitive mechanical loading leads to mitochondrial dysfunction and a decrease in ATP production in chondrocytes (Supplementary Data). Also, there is no doubt that mechanical stress-induced decreases in both mitophagy and cellular autophagy cause the accumulation of defective mitochondria and abnormal cellular proteins, resulting in reduced chondrocyte activity and affecting the maintenance of cartilage homeostasis, ultimately contributing to the progression of OA. To further analyze autophagy in greater detail, it is necessary to distinguish whether the increase in autophagosomes observed after mechanical loading in this study results from the induction of autophagy or the inhibition of autolysosome formation, and to analyze the autophagic degradation flux. In other words, to elucidate the cause of the autophagosome accumulation observed following mechanical stress, it is essential to conduct confocal fluorescence microscopy analysis using fluorescent probes that label autophagosomes and autolysosomes, in conjunction with a lysosomal acidification inhibitor. Future work will require establishing a method to observe these fluorescent probes using confocal fluorescence microscopy within a 3D culture system of chondrocytes embedded in collagen gel.

In addition, while Parkin is a key regulator of mitophagy, the process itself involves multiple steps. To substantiate conclusions regarding mitophagy impairment, further analysis, such as mitochondrial depolarization assays, analysis of mitochondrial-LC3 colocalization, and the use of mitophagy-specific reporters, is required. However, when employing measurement systems such as fluorescence microscopy, plate readers, and flow cytometry for these analyses, it proved difficult to isolate cells from 3D collagen cultures following mechanical loading while preserving their morphology. We intend to explore methods for performing these analyses on 3D collagen cultures in the future.

It is well recognized that mechanical stress directly induces excessive ROS production by mitochondria in chondrocytes, indicating a positive correlation between mechanical stress and ROS-induced oxidative stress in the pathophysiology of OA [58,59,60,61,62,63,64,65]. Mitochondria are not only ATP energy generators but are also involved in ROS generation [59]. Mechanical stress-induced mitochondrial ROS also damage mitochondria themselves; thereby, altered mitophagy caused by repetitive mechanical loading may lead to both reduced ATP production and accelerated cell damage due to oxidative stress, mediated via the accumulation of defective mitochondria in chondrocytes, consequently impairing chondrocyte function and the maintenance of articular cartilage in OA.

Our present study demonstrated that repeated compressive loading reduced protein content within cells in 3D culture after 60 or 180 min of mechanical loading, suggesting that repeated loading stress reduced chondrocyte activity, causing dead cells to detach and float from the 3D collagen. When viable cells adhering to the 3D collagen sponge were collected and analyzed for factors regulating cellular autophagy (ATG5, Beclin-1) and mitophagy (Parkin), we found that repeated compressive loading reduced the levels of autophagy regulators as a proportion of cell protein content. This suggests that under these conditions, autophagy activity was reduced, leading to the accumulation of defective proteins and mitochondrial dysfunction, which then induced cell death and detachment from the collagen sponge.

Our previous study clearly revealed that repetitive compressive loading led to the downregulation of ATP energy generation by dysfunctional mitochondria through a mechanism involving two key mitochondrial regulators, namely the sirtuin 1–NAD interaction, in chondrocytes [22]. The production levels of type II collagen and proteoglycans produced by chondrocytes did not show statistically significant differences in every individual experiment following repetitive mechanical loading, although the capacity to produce these matrix components declined markedly after loading, dropping to levels below the limit of detection in some instances. We have already found that the energy (ATP) production capacity and levels of NAD (a regulator of mitochondrial activity) following repetitive mechanical loading were altered even within a 60 min loading period (Supplementary Data). Given the time required for protein synthesis, we believe it may take a considerable amount of time for changes in intracellular mitochondrial activity and autophagy capacity to manifest as alterations in the production of cartilage matrix components (type II collagen and proteoglycans) (Supplementary Data). From our results, it has been suggested that repeated mechanical stress may alter autophagy activity, leading to the accumulation of defective proteins and mitochondrial dysfunction, thereby affecting the production of ATP by mitochondria and ultimately contributing to the progression of OA.

Over the past decade, accumulating evidence indicates the essential role of autophagy, especially mitophagy, in the pathogenesis of OA [29,30,31,32,33,34,35,36,37]. Various factors have been reported to affect autophagy activity in OA [36,37,38]. OA is a multifactorial disease characterized by the deterioration of cartilage tissue, primarily due to chondrocyte apoptosis and extracellular matrix degradation caused by various factors, including oxidative stress, mechanical stress, genomic instability, and inflammation [38]. Therefore, it has been suggested that the various factors that cause OA may affect the autophagy and mitophagy functions of chondrocytes. It has been found that autophagy- and mitophagy-related proteins including Parkin are highly expressed in articular cartilage tissues from OA patients and animal models of OA, demonstrating a strong link between mitophagy and OA [66]. Ansari et al. demonstrated that OA chondrocytes stimulated with interleukin (IL)-1 beta, which is a key mediator of OA, show overproduction of ROS, cartilage matrix collapse, accumulation of damaged mitochondria and elevated apoptosis with activation of Parkin-mediated mitophagy [29]. Furthermore, they demonstrated that depletion of Parkin in chondrocytes impairs mitophagy and gives rise to increased mitochondrial dysfunction and oxidative stress under IL-1 beta stimulation, while Parkin overexpression reduces mitochondrial ROS and chondrocyte apoptosis through the clearance of dysfunctional mitochondria [29]. These findings suggest that oxidative stress and inflammation, which are key factors in the pathogenesis of OA, reduce autophagy function in chondrocytes. Additionally, several reports demonstrated that some mitophagy-regulating compounds may function as activators of the autophagy and apoptosis signaling pathways, suppressing both oxidative stress and the imbalance between anabolism and catabolism in chondrocytes [32,34,35,36,49]. This evidence supports the important role of autophagy in maintaining chondrocyte homeostasis under OA pathology, and control of the autophagy process represents a promising option for the treatment of degenerative diseases.

In addition to oxidative stress and inflammation, mechanical stress is thought to be an important factor affecting autophagy, which is involved in the development of OA. A recent systematic review of 31 studies thoroughly examined the literature on the role of autophagy and the molecular mechanisms of OA development [67]. Of these studies, 25 investigated the effects of autophagy in aging and OA chondrocytes, and six investigated the effects of autophagy in normal human chondrocytes. Among the studies reviewed, only one study examined the effect of mechanical stress-induced autophagy on OA development in normal chondrocytes [67]. Zhang et al. demonstrated in vitro that cyclic mechanical strain with high tension induces autophagy in growth plate chondrocytes [68]. Unlike our load-bearing model for 3D-cultured chondrocytes, they applied mechanical stress using a device that applied uniaxial cyclic tensile strain to growth plate-derived chondrocytes cultured on culture plates. Their cell origin and mechanical stress application method differ from our 3D load-bearing model of articular cartilage-derived cartilage. In the present study, we demonstrated for the first time that repeated compressive loading, even within the physiological loading level, induces downregulation of regulators of both autophagy and mitophagy in chondrocytes. The repetitive mechanical stress with physiologic loading decreased production of the cartilage matrix components, proteoglycan and type II collagen, by chondrocytes. Our previous study also revealed that repetitive compressive loading led to the downregulation of ATP energy generation as a result of mitochondrial dysfunction in chondrocytes [22]. These findings suggest that a decrease in mitophagy, as well as cellular autophagy, in response to mechanical stress results in the accumulation of defective mitochondria and abnormal cellular proteins, resulting in reduced chondrocyte activity and affecting the maintenance of cartilage tissue homeostasis, ultimately contributing to the progression of OA.

Autophagy is a dynamic process, and it is crucial to determine whether the autophagy-related changes observed following mechanical loading represent a transient adaptive response or a persistent dysfunction. In a preliminary study, we examined the expression of ATG5, Beclin-1, and Parkin during a four-hour recovery period after loading. Although we have not yet reached a definitive conclusion, the trend of suppressed expression for these three factors persisted even after the four-hour recovery culture period. From a clinical perspective, joints are potentially subject to constant mechanical stress, depending on lifestyle factors. We hypothesize that stressors such as repetitive loading induce persistent changes in autophagy, rather than merely transient ones, and that this may contribute to the pathogenesis of osteoarthritis (OA). We plan to design future studies that incorporate longer recovery periods.

In conclusion, our results demonstrate that repetitive mechanical loading reduces autophagy in chondrocytes. Our findings indicate that repetitive mechanical loading reduces expression of the autophagy-regulating factors ATG5, Beclin-1 and Parkin in OA chondrocytes. We propose that a decrease in mitophagy, as well as cellular autophagy, in response to mechanical stress leads to the accumulation of defective mitochondria and abnormal cellular proteins, resulting in reduced chondrocyte activity and affecting the maintenance of cartilage tissue homeostasis, ultimately contributing to the progression of OA. Given the importance and novelty of autophagy, targeting the autophagy machinery may be a promising avenue for developing innovative therapeutic strategies. Future research should focus on translating these findings into clinically viable treatments and effectively addressing the underlying mechanisms of autophagy abnormalities in OA.

## 4. Materials and Methods

### 4.1. Monolayer Human Chondrocyte Culture

Cultured chondrocytes were isolated and established from human articular cartilage tissues of the non-weight-bearing area of surgical specimens from patients with OA (disease grade: Kellgren-Laurence grade 3 or 4), where no significant cartilage degeneration was observed in the non-weight bearing areas. The articular cartilage tissues were obtained from the knee joint of OA patients who underwent arthroplastic surgery and who provided written informed consent before participating in the study (female, 69, 71 years old, all patients’ disease grade: Kellgren-Laurence grade 4). All surgical specimens from OA patients were carefully harvested from non-weight-bearing areas, and cells were isolated exclusively from regions showing no signs of weight-bearing stress or cartilage degeneration.

The articular cartilage explants were minced into small pieces, washed with PBS, and digested individually with 1.0 mg/mL collagenase type I (Fujifilm Wako Pure Chemical Inc., Tokyo, Japan) in DMEM (Fujifilm Wako Pure Chemical Inc.) for 12 h on a shaking platform at 37 °C. Isolated chondrocytes from each explant were collected by centrifugation, washed with PBS, resuspended, and then cultured in DMEM supplemented with 10% heat-inactivated fetal calf serum, 2 mM L-glutamine, and 100 U/mL each of penicillin and streptomycin at 37 °C in a humidified atmosphere of 95% air and 5% CO_2_ as previously reported [7,22]. The study protocol was carefully reviewed and approved by the University Ethics Committee of St. Marianna University School of Medicine (permission number: 1315). The procedures followed were in accordance with the ethical standards of the Ethics Committee and with the Helsinki Declaration of 1975, as revised in 2000.

Each experiment contains cells derived from a single donor; the cells are not a mixture (pool) derived from multiple donors. Regarding each experiment, chondrocytes from a 69-year-old female were used for the first and second experiments, while chondrocytes from a 71-year-old female were used for the third and fourth experiments. Preliminary experiments confirmed no significant difference in cellular activity among chondrocytes derived from non-weight-bearing regions which were obtained from two patients, aged 69 and 71. Furthermore, a comparison of activity levels between these chondrocytes from OA patients and those from patients with joint fractures, but without OA, revealed no significant differences.

### 4.2. Generation of a 3D Cell-Collagen Scaffold Construct for Cultured Human Cells

Three-dimensional cultured tissue was generated from human chondrocytes according to the previously described method [22]. Cultured chondrocytes in a monolayer were collected and resuspended at 1.0 × 10^7^ cells/mL in DMEM (Fujifilm Wako Pure Chemical Inc., Osaka, Japan). To produce the 3D cell-collagen scaffold construct, the cell suspension was seeded onto a collagen sponge scaffold in a 96-well cell-culture plate (Iwaki AGC Techno Glass Co., Ltd., Shizuoka, Japan) at a density of 5 × 10^5^ cells/well and incubated at 37 °C in a humidified atmosphere of 95% air and 5% CO_2_. A porous collagen sponge (5 mm diameter, 3 mm thick) was purchased from AteloCell^®^ (Koken Co., Ltd., Tokyo, Japan). The pores had a mean diameter of approximately 100 µm (range from 50 to 200 µm). After generation of the 3D cell–collagen scaffold construct, the 3D culture tissues were incubated for 2 weeks in a growth medium for chondrocytes, as appropriate, at 37 °C in a 5% CO_2_ atmosphere.

### 4.3. Repetitive Compressive Loading of the 3D Cell-Culture Construct

The 3D cell–collagen scaffolds described above were placed into individual wells of a 24-well culture dish (AGC Techno Glass) and maintained in 0.7 mL DMEM. Then, repetitive compressive loading was applied to the 3D cell–collagen scaffolds using a custom-designed and built apparatus as shown in Figure 2. A physiologic and compressive load was repetitively applied to the test 3D constructs at 0 kPa (non-loaded) or 25.5 gf/cm^2^ (2.5 kPa) [22].

The repetitive compressive loading experiments were carried out for 60 or 180 min at a rate of 52 cycles/min in a humidified incubator maintained at 37 °C in a 5% CO_2_ atmosphere. 3D constructs without loading were used as the control. After the incubation period, culture-conditioned medium and cellular proteins were collected and stored at −80 °C until they were used for analysis.

### 4.4. Cellular Protein Content in the 3D Cell-Culture Construct

The total protein content of the cells in each 3D cell–collagen sponge construct was extracted by the following method [7,22,51]. Cell protein contents were collected from the 3D collagen sponges immediately after repeated loading. After washing the 3D cell–collagen scaffolds three times with PBS, 150 µL of collagenase and protein extraction reagent (T-PER^TM^: Thermo Fisher Scientific, Waltham, MA, USA) containing protease inhibitors (cOmplete^TM^: Merck Millipore, Darmstadt, Germany) was added, and the samples were finely chopped. The separated cells were recovered by sonication and gentle centrifugation. These were further centrifuged (15,000 rpm, 4 °C, 5 min) and recovered for in vitro analysis. Protein samples were collected from the cells for subsequent analysis.

The collagen sponge was completely enzymatically degraded and dissolved by collagenase; the sponge structure disappeared, transforming the material into a solution. It is considered that the viable cells adhering to the 3D collagen sponge structure were recovered without remaining within the sponge matrix.

### 4.5. Western Blot Assay

To study the effects of repetitive compressive loading on the expression of Parkin, ATG5 and Beclin-1 in chondrocytes, the levels of these three proteins in cells were analyzed by Western blotting as previously described [7,22]. For Western blotting, the antibodies used were a monoclonal antibody against human Parkin (1:1000 dilution; Santa Cruz Biotechnology, Inc., Santa Cruz, CA, USA), a polyclonal antibody against human ATG5 (1:1000 dilution) and Beclin-1 (1:1000 dilution) (both from Cell Signaling Technology, Inc., Danvers, MA, USA), and the corresponding secondary antibody conjugated with horseradish peroxidase (HRP) [anti-Mouse Ig conjugated with HRP for anti-Parkin antibody (1:5000 dilution; DAKO A/S, Glostrup, Denmark), or anti-Rabbit Ig conjugated with HRP for anti-ATG5 or anti-Beclin-1 antibody (1:4000 dilution; DAKO A/S)] and used anti-beta-actin antibody conjugated with HRP Mouse-monoclonal (1:10,000 dilution; Proteintech Group, Inc., Rosemont, IL, USA) as a loading control for Western blotting.

Seven micrograms of cellular protein were loaded onto each lane of a 10% sodium dodecyl sulfate polyacrylamide gel and subjected to electrophoresis (SDS-PAGE) followed by Western blotting. The antibody-bound protein bands were visualized, and the densitometry of the signal bands was analyzed using an extended cavity laser system (GE Healthcare Bio-sciences KK, Tokyo, Japan).

### 4.6. Effects of Repetitive Mechanical Loading on Cellular Activities In Vitro

In chondrocytes, to verify the involvement of mechanical loading in each cellular activity under different conditions of cell strain, we analyzed the level of each cell strain-specific factor after mechanical loading of the 3D cell-collagen sponge construct. Cellular proteins and conditioned medium were collected from the 3D culture construct, and the levels of proteoglycan and type II collagen produced by chondrocytes were measured using ELISA kits (proteoglycan assay kit; Thermo Fisher Scientific Inc.; type II collagen assay kit; Fine Test, Wuhan, China) and an ELISA plate reader at 450 nm (Multiskan™ FC Microplate Photometer, Thermo Fisher Scientific Inc.).

### 4.7. Scanning Electron Microscopy

Transmission Electron Microscopy (TEM) analysis was performed to examine the surface structure of the 3D culture constructs, after the treatment of the 3D cell-culture scaffold with the repetitive compressive loading or non-loading (control). After serial fixations of scaffold samples with 2% glutaraldehyde, 1% osmium tetroxide, and 1% tannic acid aqueous solution, the surface morphology of the 3D cell-culture scaffolds was observed by TEM (S-4800, Hitachi Ltd., Tokyo, Japan).

In each group, the numbers of autophagosomes, lysosomes, and autolysosomes per cell were measured for 10 cells.

## Data Availability

The original contributions presented in this study are included in the article/Supplementary Materials. Further inquiries can be directed to the corresponding author.

## Supplementary Materials

The following supporting information can be downloaded at: https://www.mdpi.com/article/10.3390/ijms27167485/s1.
