# Supplementary material for: Repetitive Compressive Loading Downregulates the Expression of Autophagy-Related Factors, Autophagy Capacity and Cellular Activity in Human Osteoarthritic Chondrocytes
Source: Int J Mol Sci. 2026 Aug 21;27(16):7485. doi: 10.3390/ijms27167485 (PMC13513576; doi:10.3390/ijms27167485)
Supplement: Supplementary file 1 [file ijms-27-07485-s001.zip › ijms-4424863-supplementary.pdf]

Supplementary data

Figure S1.

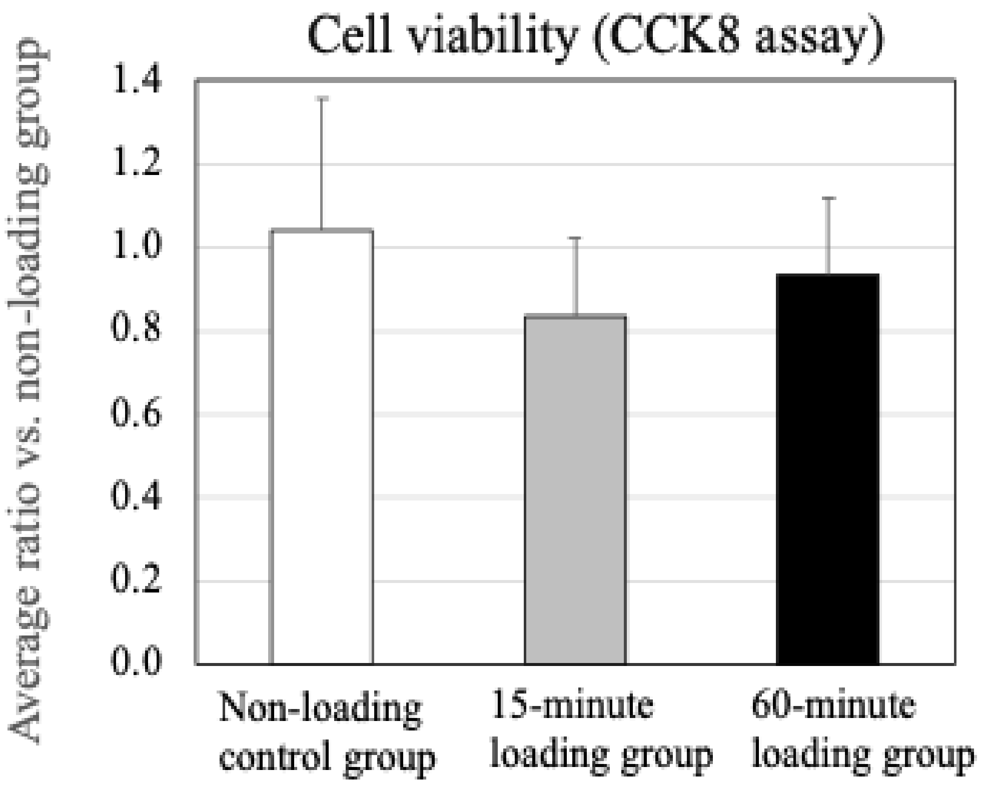

Figure S1. Effects of physiologic and repetitive compressive loading on chondrocyte viability in the 3D cell-culture construct after mechanical loading.

Cell viability was analyzed using the CCK-8 assay. A load of 2.5 kPa did not adversely affect cell viability.

Figure S2.

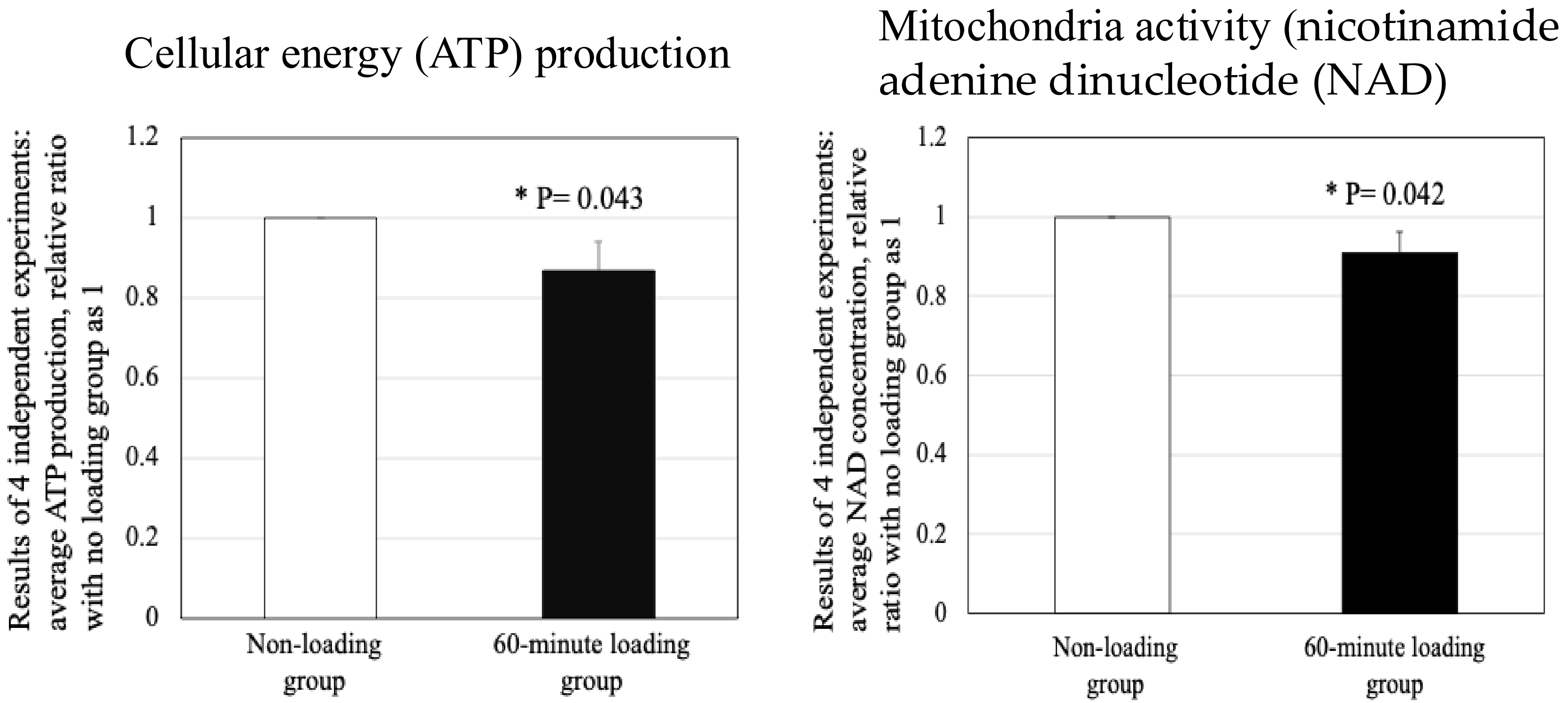

Figure S2. Effects of physiological and repetitive compressive loading on ATP production and nicotinamide adenine dinucleotide (NAD) activity in chondrocytes.

Significant differences in ATP production and NAD activity in chondrocytes were observed between the unloaded control group and the group subjected to 60 minutes of loading.
